# CCZ1 Accelerates the Progression of Cervical Squamous Cell Carcinoma by Promoting MMP2/MMP17 Expression

**DOI:** 10.3390/biomedicines12071468

**Published:** 2024-07-03

**Authors:** Jing Yu, Zhenlong Yuan, Jing Liu, Lu Deng, Yuting Zhao, Shengnan Wang, Enyu Tang, Xi Yang, Ning Li, Jusheng An, Lingying Wu

**Affiliations:** 1 Department of Gynecology Oncology, National Cancer Center/National Clinical Research Center for Cancer/Cancer Hospital, Chinese Academy of Medical Sciences and Peking Union Medical College, Beijing 100021, China; 17611768237@126.com (J.Y.); yzlbetablocker@gmail.com (Z.Y.); liujinglnnu@163.com (J.L.); 18217721131@163.com (L.D.); b2023003100@pumc.edu.cn (Y.Z.); 13840598176@163.com (S.W.); b2023003101@pumc.edu.cn (E.T.); yangxi_pumc@outlook.com (X.Y.); lining1502@csco.ac.cn (N.L.); 2 State Key Laboratory of Molecular Oncology, National Cancer Center/National Clinical Research Center for Cancer/Cancer Hospital, Chinese Academy of Medical Sciences and Peking Union Medical College, Beijing 100021, China

**Keywords:** CCZ1, cervical squamous cell carcinoma, MMP2, MMP17

## Abstract

Cervical squamous cell carcinoma (CSCC) represents a significant global health concern among females. Identifying new biomarkers and therapeutic targets is pivotal for improving the prognosis of CSCC. This study investigates the prognostic relevance of CCZ1 in CSCC and elucidates its downstream pathways and targets using a combination of bioinformatics analysis and experimental validation. Transcriptomic analysis of 239 CSCC and 3 normal cervical samples from The Cancer Genome Atlas database reveals a marked upregulation of CCZ1 mRNA levels in CSCC, and elevated CCZ1 mRNA levels were associated with poor prognosis. Immunohistochemical analysis of clinical samples also confirmed these findings. Furthermore, functional assays, including Cell Counting Kit-8, colony formation, Transwell, and flow cytometry, elucidated the influence of CCZ1 on CSCC cell proliferation, migration, invasion, and cell cycle progression. Remarkably, CCZ1 knockdown suppressed CSCC progression both in vitro and in vivo. Mechanistically, CCZ1 knockdown downregulated MMP2 and MMP17 expression. Restoring MMP2 or MMP17 expression rescued phenotypic alterations induced by CCZ1 knockdown. Hence, CCZ1 promotes CSCC progression by upregulating MMP2 and MMP17 expression, emerging as a novel biomarker in CSCC and presenting potential as a therapeutic target in CSCC.

## 1. Introduction

Cervical cancer (CC) is one of the most prevalent malignancies in females globally. It contributes to more than 500,000 new cases and 300,000 fatalities annually [1,2]. Cervical squamous cell carcinoma (CSCC) constitutes most cases, accounting for 75% to 90%, and cervical adenocarcinoma (CAde) comprises 10% to 25% of cases [3]. The initiation and progression of CSCC, which progresses from cervical intraepithelial neoplasia (CIN) to early and advanced stages, are intricately, but not exclusively, associated with chronic human papillomavirus (HPV) infection [4,5]. Although the HPV vaccine has mitigated the incidence and mortality of CC in high-income countries, it is a pressing social concern that severely jeopardizes women’s health in low-income nations [6]. There is limited understanding of the molecular mechanisms underlying cervical cancer development. Patients with early stage cervical cancer who undergo surgery typically have a favorable prognosis, but patients with locally advanced disease often rely on concurrent radiotherapy and chemotherapy, which leads to a less optimistic prognosis [7]. The lack of clinical biomarkers capable of prognostic prediction is evident. The screening and identification of dependable biomarkers and drug treatment targets hold significant potential for advancing personalized cervical cancer therapy [8]. These endeavors have crucial practical implications for enhancing patient survival rates and quality of life.

CCZ1 is also known as vacuolar fusion protein CCZ1 homolog. It functions with MON1A as a guanine exchange factor (GEF) for RAB7 (RAB7, member RAS oncogene family). Together, they help convert RAB7 from its inactive guanosine diphosphate (GDP)-bound state to its active guanosine triphosphate (GTP)-bound form by promoting GDP-to-GTP exchange [9,10,11]. CCZ1 participates in various intracellular material transport processes, including autophagy and endosomal maturation [12,13,14]. CCZ1 alleviated neuropathology and memory defects in Alzheimer’s disease models by enhancing autophagy maturation [12]. However, there is a dearth of studies on the association between CCZ1 expression and tumorigenesis. Bioinformatics screening revealed that elevated CCZ1 mRNA levels in CSCC were an independent risk factor for a poor prognosis. Therefore, investigating the role and function of CCZ1 in CSCC provides novel experimental evidence for elucidating the mechanisms underlying the malignant progression of CSCC and potentially facilitates the advancement of personalized treatment approaches for this disease.

Matrix metalloproteinases (MMPs), also known as matrix metallopeptidases or matrixins, are a family of calcium-dependent zinc-containing endopeptidases within the metzincin superfamily of proteases [15,16]. These enzymes degrade a wide array of extracellular matrix proteins and are involved in the processing of various bioactive molecules [17]. MMPs play pivotal roles in numerous cellular processes, including the cleavage of cell surface receptors [18,19], the release of apoptotic ligands, such as FAS ligands [20], and the inactivation of chemokines and cytokines [21]. MMPs are also implicated in fundamental cell behaviors, such as proliferation, migration, differentiation, angiogenesis, apoptosis, and host defense mechanisms [22,23,24]. Mounting evidence suggests that dysregulation of MMPs, particularly MMP2 and MMP17, plays a pivotal role in tumor invasion, metastasis, and the microenvironment in various cancer types [25,26]. MMP2 and MMP17 have been implicated in extracellular matrix (ECM) degradation, angiogenesis, and the facilitation of tumor cell migration and invasion, which contribute to disease progression and poor patient outcomes [27,28,29]. However, the roles of MMPs in the progression of CSCC and the relationship between CCZ1 and MMP2/17 are not clear.

Recent advances in targeted therapy for cervical cancer have shown promising results, with several studies focusing on molecular targets such as epidermal growth factor receptor (EGFR), vascular endothelial growth factor (VEGF), and programmed death-1 (PD-1)/programmed death-ligand 1(PD-L1) pathways [30,31,32]. These therapies aim to improve patient outcomes by targeting specific molecular mechanisms involved in cancer progression. Despite these advancements, there remains a critical need for identifying new prognostic markers and therapeutic targets to enhance treatment efficacy and patient survival. The present study elucidated the functional significance of CCZ1 in CSCC progression and its mechanistic association with MMP2 and MMP17 expression. Our analysis demonstrated that CCZ1 was overexpressed in CSCC tumor tissues and was an independent prognostic risk factor affecting the overall survival (OS) of CSCC patients. It promoted CSCC cell proliferation, colony formation, migration, and invasion by upregulating MMP2 and MMP17 expression. These findings elucidate the intricate molecular networks driving CSCC progression. This study highlights the importance and clinical need for developing novel therapeutic strategies targeting CCZ1 and MMPs of CSCC patients, ultimately improving their prognosis and treatment outcomes.

## 2. Materials and Methods

### 2.1. Data Collection and Processing

CSCC samples with complete prognostic data were used. Clinical information and RNA sequencing (RNA-Seq) data for 239 CSCC samples and 3 normal cervical tissue samples were obtained from The Cancer Genome Atlas (TCGA) via the University of California, Santa Cruz (UCSC) Xena Browser (http://xena.ucsc.edu/, accessed on 29 March 2023). Gene expression levels were normalized as fragments per kilobase of transcript per million mapped reads (FPKM) then log_2_-transformed. Notably, all patients included in the dataset had no prior history of other malignant tumors or diseases, and none had received adjuvant treatment before surgery.

### 2.2. Tissue Samples

Between September 2018 and December 2021, 7 paired samples of CSCC tissues and normal cervical tissues were procured from patients who underwent surgical resection at the National Cancer Center/National Clinical Research Center for Cancer/Cancer Hospital, Chinese Academy of Medical Sciences and Peking Union Medical College. After surgical excision, pathologists promptly isolated tissue from each patient and preserved samples in liquid nitrogen for RNA analysis. Two pathologists validated the pathological examination of CSCC samples. Additionally, Formalin-fixed Paraffin-embedded (FFPE) samples of 80 CSCC tissues and 8 normal cervical tissues were obtained from the National Cancer Center/National Clinical Research Center for Cancer/Cancer Hospital, Chinese Academy of Medical Sciences and Peking Union Medical College. The information of the cohort is listed in Table S1. Ethical approval was obtained from the Ethics Committee of the National Cancer Center/National Clinical Research Center for Cancer/Cancer Hospital, Chinese Academy of Medical Sciences and Peking Union Medical College (24/308-4588).

### 2.3. Differential Expression and Prognosis Correlation Analysis

The clinical phenotypes were categorized and analyzed using the R package “limma” to compute the differential expression of CCZ1 between various clinical subgroups. Based on the median CCZ1 expression, CSCC patients were stratified into CCZ1-high and CCZ1-low-expression groups. Survival plots were generated using the R packages “survivor” and “survminer”, and box plots were created using the R package “ggplot2”. Differential expression analysis of CCZ1 in cholangiocarcinoma (CHOL), glioblastoma multiforme (GBM), pancreatic adenocarcinoma (PAAD), thymoma (THYM), kidney renal papillary cell carcinoma (KIRP), and lymphoid neoplasm diffuse large B-cell lymphoma (DLBC) was performed using GEPIA (http://gepia.cancer-pku.cn/, accessed on 6 January 2024) [33]. All analyses were performed using the R Foundation for Statistical Computing (2024) version 4.4.0.

### 2.4. Construction and Validation of the Nomogram

We performed univariate and multivariate Cox regression analyses to examine the association between CCZ1 levels and CSCC prognosis. For more accurate prognostic estimations, we constructed a nomogram that incorporated CCZ1 levels and clinical factors using the R package “rms”. Calibration plots were generated to evaluate the agreement between the observed survival rates and the rates predicted by the nomogram to assess its calibration performance.

### 2.5. Functional Enrichment Analysis

We used a correlation coefficient threshold greater than 0.3 to identify CCZ1-related genes. Using these genes, we performed gene set enrichment analysis (GSEA) using the R packages “GSEABase”, “DESeq2”, “clusterProfiler”, and “enrichplot”. The cutoffs for significance were set at a |normalized enrichment score (NES) > 1, nominal (NOM) *p* value < 0.05, and false discovery rate (FDR) q value < 0.25. Gene Ontology (GO) analysis and Kyoto Encyclopedia of Genes and Genomes (KEGG) pathway analysis were performed using the “clusterprofiler” package to elucidate the functional roles of CCZ1. Differentially expressed genes in the high-CCZ1- and low-CCZ1-expression groups were identified based on the criterion of “Log_2_|fold change| > 1, *p* < 0.05”.

### 2.6. Cell Culture and Transfection

The human CSCC cell lines ME180 and SiHa were obtained from the Cell Resource Center, Peking Union Medical College (PCRC). ME180 is a cell line with epidermoid carcinoma (a highly invasive squamous cell carcinoma). SiHa is a cell line with squamous cell carcinoma. ME180 cells were cultured in McCoy’s 5A medium (Gibco, Grand Island, NY, USA), and SiHa cells were cultured in minimum essential medium α (MEMα) (Gibco, Grand Island, NY, USA) supplemented with 10% fetal bovine serum (Invitrogen, San Diego, CA, USA) at 37 °C with 5% CO_2_. Small interfering RNA (siRNA) and non-silencing siRNA controls were synthesized by GenePharma (Shanghai, China) and transfected using Lipofectamine 2000 (Invitrogen, San Diego, CA, USA) at a final concentration of 50 nM following the manufacturer’s instructions. The MMP2 and MMP17 expression vectors containing human MMP2 or MMP17 coding sequences in the PLVX-puro expression vector were obtained from GenePharma (Shanghai, China) and transiently transfected using Lipofectamine 2000 according to the manufacturer’s instructions. The lentiviral vector PLKO.1-shCCZ1-GFP, which was used to knock out CCZ1, was constructed by GenePharma. The lentivirus was incubated with polybrene (Sigma, St. Louis, MO, USA) and transfected into CSCC cells, followed by puromycin selection (0.8 mg/mL, Invitrogen) for 72 h. The sequences of the siRNAs and short hairpin RNAs (shRNAs) used are listed in Table S2.

### 2.7. Cell Viability Assay

Cells were plated in triplicate in 96-well plates at a density of 2000 cells per well. Cell viability was assessed using a Cell Counting Kit-8 (CCK-8, Dojindo, Japan). Cells were adhered for 7 consecutive days according to the manufacturer’s protocol. The absorbance at 450 nm was quantified using an ELX808 microplate spectrophotometer (BioTek Instruments, Winooski, VT, USA).

### 2.8. Colony Formation Assay

For in vitro assessment of cell colony formation capacity, 500 cells were seeded in triplicate in 6-well culture plates and cultured for 10–14 days until visible colonies emerged. These colonies were fixed with methanol, stained with crystal violet, and quantified.

### 2.9. Cell Cycle Analysis

The cell cycle distribution of 1.0 × 10^6^ CCZ1-knockdown cells and 1.0 × 10^6^ negative control cells was analyzed as follows. Cells were fixed with 70% ethanol at 4 °C overnight, resuspended, and analyzed using a cell cycle detection kit (KeyGen Biotech, Nanjing, China) and BD™ LSRII flow cytometry (BD Biosciences, San Jose, CA, USA). Cell cycle profiles were analyzed using ModFit LT software (Version 5.0.9) (Verity Software House, Topsham, ME, USA).

### 2.10. Transwell Migration/Invasion Assays

For the invasion and migration assays, Transwell inserts (Corning, NY, USA) were used. Matrigel (Corning, NY, USA) was applied to the bottom center of the upper chamber and incubated at 37 °C for 5 h for the invasion assay, with excess liquid removed. Matrigel was not used for the migration assay. In the upper chamber, 1 × 10^5^ cells were seeded in 100 µL of serum-free medium containing 0.1% bovine serum albumin (Applygen, Beijing, China). The lower chamber was filled with 500 µL of medium containing 10% fetal bovine serum. After 24 h of incubation, the upper chamber was fixed with 4% paraformaldehyde (Applygen, Beijing, China) for 15 min at room temperature. The upper chamber was stained with crystal violet staining solution (Applygen, Beijing, China) for 20 min, dried, and imaged under a microscope. Five randomly selected fields were photographed at 10× magnification. ImageJ software (Version 1.54i) was used for cell number quantification. Each assay was performed in triplicate.

### 2.11. Total RNA Extraction, Quantitative Real-Time PCR (qRT-PCR)

Total RNA was extracted following the manufacturer’s instructions using an RNApure Tissue & Cell Kit (Cwbiotech, Beijing, China). Subsequently, the isolated RNA served as a template for reverse-transcription reactions employing a HiFiScript cDNA Synthesis Kit (Cwbiotech, Beijing, China). qRT-PCR analysis was conducted using SYBR^®^ Fast qPCR Mix (TaKaRa, Shiga, Japan) and a CFX96 Real-Time System (Bio-Rad, Berkeley, CA, USA). The primer sequences utilized are detailed as follows: GAPDH: 5′-GTCTCCTCTGACTTCAACAGCG-3′ (Forward) and 5′-ACCACCCTGTTGCTGTAGCCAA-3′ (Reverse), CCZ1: 5′-TTGCCGAAGACTGGACAGCATC-3′ (Forward) and 5′-TGTGCTCTTCTCGGCGAGATTC-3′ (Reverse). Each sample was independently analyzed three times.

### 2.12. Western Blot Analysis

Protein lysates were resolved on SDS-PAGE gels and transferred to PVDF membranes (Millipore, Bedford, MA, USA). Membranes were blocked and incubated with primary antibodies targeting CCZ1 (Proteintech, Wuhan, Hubei, China), MMP2 (Proteintech, Wuhan, Hubei, China), MMP17 (Proteintech, Wuhan, Hubei, China), and cyclin B1 (Cell Signaling Technology, Danvers, MA, USA). The membranes were probed with the corresponding secondary antibodies. GAPDH (Proteintech, Wuhan, Hubei, China) served as the loading control. Signal visualization was achieved using a super-enhanced chemiluminescence detection reagent (Applygen, Beijing, China).

### 2.13. Tumor Xenograft Model

All animal procedures were performed according to the guidelines approved by the National Cancer Center/National Clinical Research Center for Cancer/Cancer Hospital, Chinese Academy of Medical Sciences and Peking Union Medical College (NCC2021A-078) and adhered to the National Institutes of Health Guide for the Care and Use of Laboratory animals. SiHa-shscramble and SiHa-shCCZ1 cells (2 × 10^6^) were subcutaneously injected into both experimental groups (*n* = 4). The mice were weighed, and the tumor size was measured twice weekly. The tumor volume (V) was calculated using the formula V = 0.524 × L × W^2^, where L represents the length, and W denotes the width of the xenograft tumor. Three weeks after treatment, the mice were euthanized, and the average weight of the tumor tissues was determined.

### 2.14. Immunohistochemistry (IHC) and Quantification

A total of 80 CSCC tissues and 8 normal cervical tissues FFPE samples were obtained from patients who underwent treatment at the National Cancer Center/National Clinical Research Center for Cancer/Cancer Hospital, Chinese Academy of Medical Sciences and Peking Union Medical College between September 2018 and December 2021. For immunohistochemistry, 4 mm thick sections of paraffin-embedded tissue arrays were deparaffinized in xylene, rehydrated in graded ethanol, and incubated in 3% hydrogen peroxide in methanol to block endogenous peroxidase. The sections were subjected to heat treatment for 20 min in 1 mM of EDTA buffer (pH 8.0) using a microwave oven. The sections were washed three times with PBS and incubated overnight at 4 °C with an anti-human CCZ1 antibody (1:200 dilution, Proteintech, Wuhan, Hubei, China). After washing with PBS, the sections were developed according to the manufacturer’s instructions (PV-9000 Polymer Detection System, Zhongshan Golden Bridge, Beijing, China), counterstained with hematoxylin, dehydrated in graded ethanol, and sealed with neutral resin. Two investigators independently evaluated the immunohistochemical (IHC) staining of CCZ1 on the slides. The slides were scanned using Aperio ScanScope XT system (Leica Biosystems, IL, USA), and the images were magnified 40 times for histological scoring. A blinded assessment of the immunohistochemistry (IHC) images was performed using IHC Profiler, an ImageJ plug-in, following previously described methods [34]. Immunostaining in specific specimen areas was classified as negative (−), slightly positive (+), moderately positive (++), or strongly positive (+++). The CCZ1 H-score was determined using the following linear equation: H-score = 1 × (% slightly positive cells) + 2 × (% moderately positive cells) + 3 × (% strongly positive cells), as described in previous studies [35].

### 2.15. RNA-Seq

Total RNA was extracted from SiHa non-silencing cells and SiHa-CCZ1-siRNA cells, and the integrity of the extracted RNA was assessed using agarose gel electrophoresis. The concentration of RNA was determined using a NanoDrop spectrophotometer (Thermo Fisher Scientific, Waltham, MA, USA). Library construction and quality control procedures were performed. Sequencing was performed using a next-generation sequencing system. At least three biological replicates were performed for each group. RNA-Seq and subsequent analyses were performed by Shanghai Mingma Technology Co., Ltd. (Shanghai, China).

### 2.16. Statistical Analysis

All statistical analyses were performed using SPSS 29.0.0 software (SPSS Inc., Chicago, IL, USA). Statistical significance was defined as a probability value of *p* < 0.05. Kaplan–Meier analysis with the log-rank test was used to assess differences in OS and progression-free survival (PFS) between groups. Student’s t test was used to determine the significance of differences between two groups, and one-way ANOVA was used for comparisons of more than two groups.

## 3. Results

### 3.1. CCZ1 mRNA Levels Were Elevated in CSCC Tissues, and Increased CCZ1 mRNA Levels Correlated with Poor Prognosis

**Figure 1:**
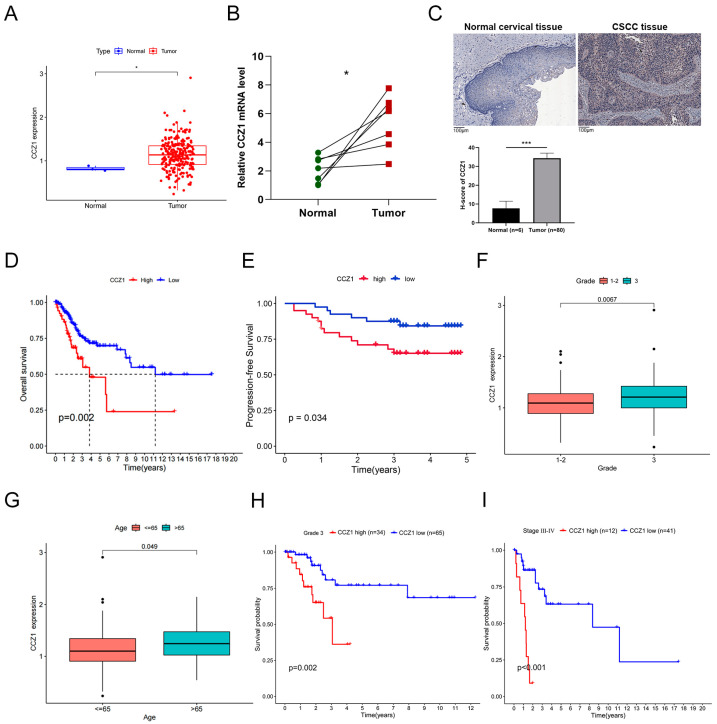
CCZ1 mRNA was elevated in CSCC tumor tissues, and increased CCZ1 mRNA levels correlated with poor prognosis. (**A**) Abnormally high CCZ1 mRNA levels in CSCC. (**B**) qRT-PCR analysis of CCZ1 mRNA relative level in paired samples of CSCC tissues and normal cervical tissues (*n* = 7). (**C**) IHC analysis of CCZ1 protein expression in normal cervical tissues and CSCC tissues. (**D**) The OS curves of the CCZ1-high group and CCZ1-low group sourced from TCGA database. (**E**) The PFS curves of the CCZ1-high group and CCZ1-low group sourced from clinical patients. (**F**) Association of the CCZ1 mRNA level with age. (**G**) Association of the CCZ1 mRNA level with tumor grade. (**H**) The Kaplan–Meier survival curves of the CCZ1-high and CCZ1-low groups in the grade 3 subgroup. (**I**) The Kaplan–Meier survival curves of the CCZ1-high and CCZ1-low groups in the FIGO stage Ⅲ-Ⅳ subgroup. * *p* < 0.05, *** *p* < 0.001. CSCC: cervical squamous cell carcinoma; qRT-PCR: quantitative real-time PCR; IHC: immunohistochemistry; TCGA: The Cancer Genome Atlas; OS: overall survival; PFS: progression-free survival; FIGO: International Federation of Gynecology and Obstetrics.

We compared the CCZ1 mRNA levels in normal cervical tissue samples to CSCC tissue samples from TCGA datasets. CCZ1 was significantly overexpressed in CSCC tissue (*n* = 239) compared to normal cervical tissues (*n* = 3) (*p* < 0.05, Figure 1A). We investigated the expression levels of CCZ1 mRNA in other tumor types. Our findings revealed a notable increase in CCZ1 expression in tumor tissues compared to normal tissues in CHOL (*p* < 0.05, Figure S1A), GBM (*p* < 0.05, Figure S1B), PAAD (*p* < 0.05, Figure S1C), THYM (*p* < 0.05, Figure S1D), KIRP (*p* < 0.05, Figure S1E), and DLBC (*p* < 0.05, Figure S1F) patients. Subsequently, qRT-PCR analysis was performed on paired samples of CSCC tissues and normal cervical tissues (*n* = 7), revealing a significant upregulation of CCZ1 mRNA in CSCC tissues (*p* < 0.05, Figure 1B). Representative IHC images in Figure 1C show a notable increase in CCZ1 expression in CSCC tissues (*n* = 80) compared to normal cervical tissues (*n* = 6) (*p* < 0.001). To further explore the association between CCZ1 expression and the prognosis of CSCC patients, we stratified 239 CSCC patients into two groups, 62 patients with high CCZ1 expression and 167 patients with low CCZ1 expression, based on median CCZ1 expression. The Kaplan−Meier survival curves indicated that patients with high CCZ1 levels had a lower OS (*p* = 0.002, Figure 1D). In addition, we conducted immunohistochemical staining on tumor tissues obtained from 80 patients diagnosed with CSCC. Subsequently, the cohort was stratified into two groups based on CCZ1 expression: high (*n* = 40) and low (*n* = 40), using the median expression value as the cutoff. Kaplan–Meier survival analysis revealed a significant association between high CCZ1 expression and poorer PFS (*p* = 0.034, Figure 1E). These findings suggest a potential prognostic relevance of CCZ1 expression in CSCC, wherein elevated expression levels correlate with adverse prognosis. Our analysis revealed significant differences in CCZ1 mRNA levels between subgroups in age (*p* = 0.049, Figure 1F) and grade (*p* = 0.0067, Figure 1G). The Kaplan–Meier survival curves of the grade 3 subgroup and the International Federation of Gynecology and Obstetrics (FIGO) stage III–IV subgroup demonstrated that patients with high CCZ1 levels exhibited a poorer prognosis (*p* = 0.002, *p* < 0.001, respectively, Figure 1H,I). These results suggest that CCZ1 is significantly highly expressed in CSCC samples and correlates with poor prognosis across multiple clinical subgroups. This finding supports the potential of CCZ1 as a promising biomarker for predicting the prognosis of CSCC patients.

### 3.2. Establishment and Validation of a Nomogram Based on CCZ1

**Figure 2:**
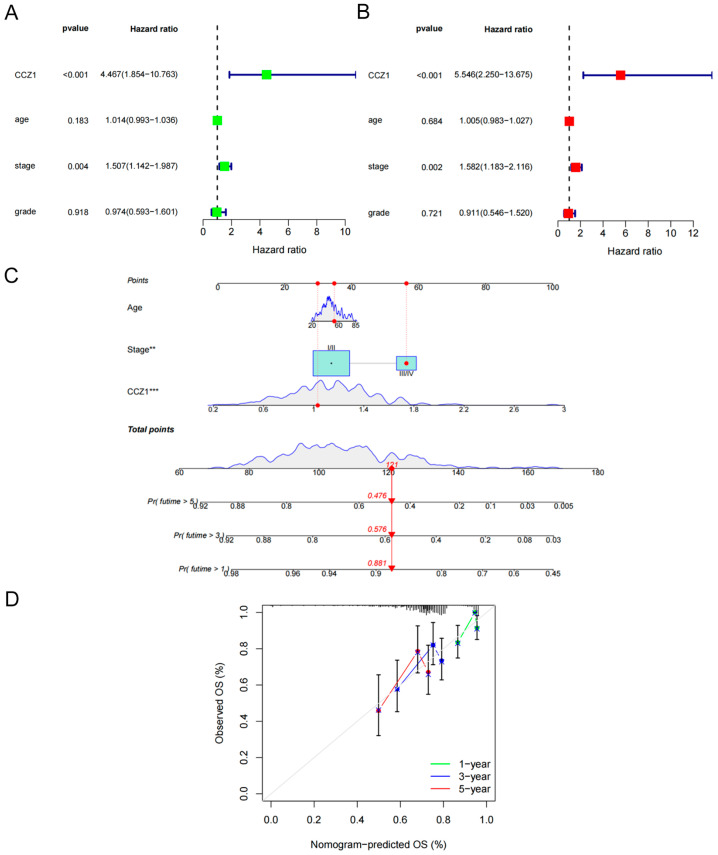
Establishment and validation of a nomogram based on CCZ1. (**A**) Univariate Cox regression analysis. (**B**) Multivariate Cox regression analysis. (**C**) A nomogram was constructed to predict the 1-, 3- and 5-year survival rates of CSCC patients. (**D**) Calibration curves showing the actual rate versus the predicted probability of 1-, 3- and 5-year survival. CSCC: cervical squamous cell carcinoma. ** *p* < 0.01, *** *p* < 0.001.

We performed univariate and multivariate Cox regression analyses to evaluate the independent prognostic significance of clinicopathological parameters (age, stage, and grade) and CCZ1 levels derived from the TCGA dataset (Figure 2A,B). According to the multivariate Cox regression analysis, the hazard ratio (HR) for CCZ1 was 5.546 (*p* < 0.001, Figure 2B), which indicated that CCZ1 was an independent risk factor for the prognosis of CSCC patients. To develop an accurate prognostic classifier for CSCC patients, we devised a nomogram integrating age, stage, and CCZ1 levels (Figure 2C). The calibration curves for the 1-, 3-, and 5-year survival rates closely matched the standard curves, which indicated excellent predictive performance of the nomogram (Figure 2D).

### 3.3. Association of CCZ1 with Immune Checkpoints and Immunogenic Cell Death Modulators

**Figure 3:**
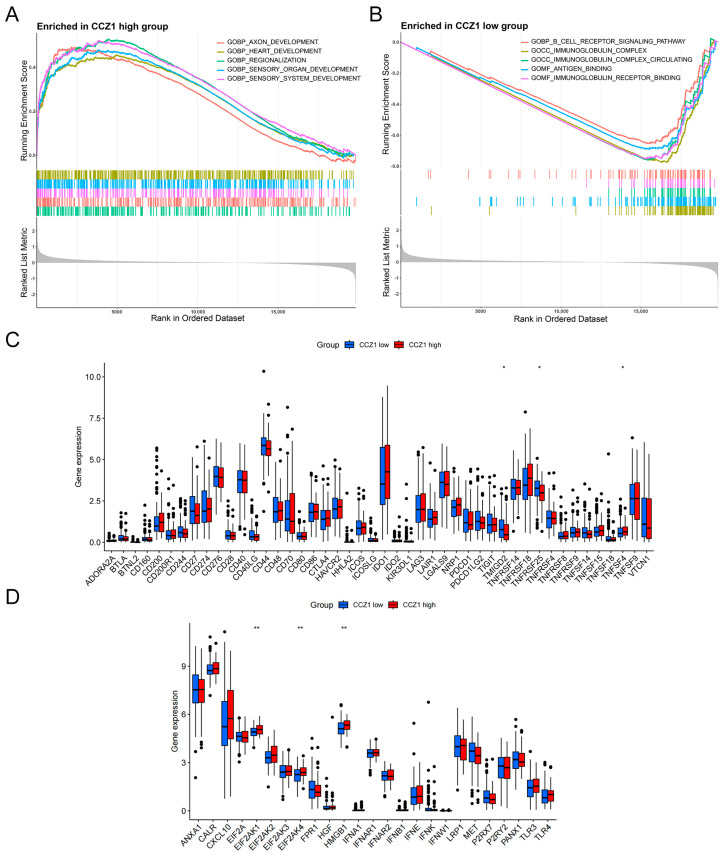
Association of CCZ1 with immune checkpoints and immunogenic cell death modulators. (**A**) GSEA of gene sets that were significantly enriched in the CCZ1-high group. (**B**) GSEA of gene sets that were significantly enriched in the CCZ-low group. (**C**) Association of the CCZ1 mRNA level with the levels of immune checkpoint genes. (**D**) Association of the CCZ1 mRNA level with the level of immunogenic cell death-related molecules. * *p* < 0.05, ** *p* < 0.01. GSEA: Gene Set Enrichment Analysis.

GSEA was performed for CCZ1-related gene signatures. The results revealed significant enrichment of gene signatures associated with various developmental processes in the high-CCZ1-expression group, such as axon development, heart development, regionalization, sensory organ development, and sensory system development (Figure 3A). Conversely, gene signatures related to the B cell receptor signaling pathway, immunoglobulin complex, circulating immunoglobulin complex, antigen binding, and immunoglobulin receptor binding were significantly enriched in the low-CCZ1-expression group (Figure 3B). These results suggest that reduced CCZ1 expression is associated with immune system-related processes. Previous research established the pivotal involvement of immune checkpoints and immunogenic cell death modulators in the regulation of host anti-tumor immunity [36]. Therefore, the comparative expression of these modulators was evaluated in the two subgroups. We investigated the correlation between CCZ1 expression and the expression of immune checkpoints and immunogenic cell death modulators in CSCC. We observed significant upregulation of the immune checkpoints TMIGD2, TNFRSF25, and TNFSF4 in the high-CCZ1-expression group (*p* < 0.05, *p* < 0.05, *p* < 0.05, respectively; Figure 3C). The levels of the immunogenic cell death modulators EIF2AK1, EIF2AK4, and HMGB1 were significantly greater in the high-CCZ1-expression group compared to the low-CCZ1-expression group (*p* < 0.01, *p* < 0.01, *p* < 0.01, respectively; Figure 3D). These results suggest that CCZ1 expression is associated with various immune checkpoints and immunogenic cell death modulators, which supports its potential involvement in the immune regulation of CSCC.

### 3.4. CCZ1 Knockdown (KD) Inhibited CSCC Progression In Vitro and In Vivo

**Figure 4:**
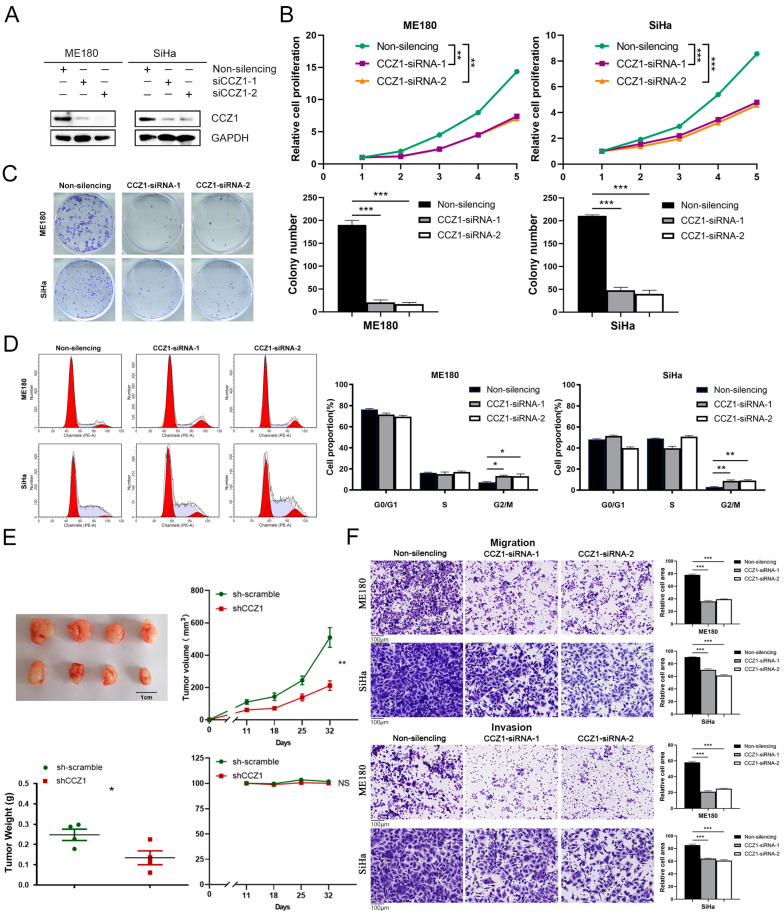
CCZ1 KD inhibited CSCC progression in vitro and in vivo. (**A**) Western blotting analysis of CCZ1 expression in ME180 cells and SiHa cells transfected with CCZ1 siRNA or non-silencing siRNA. (**B**) Cell proliferation was detected using the CCK-8 assay. (**C**) Cell colony formation assay. (**D**) Flow cytometric analysis of the cell cycle. (**E**) Xenograft tumors, tumor growth curves, tumor weights, and body weights of the mice. (**F**) Transwell assay for migration and invasion capability analysis. * *p* < 0.05, ** *p* < 0.01, *** *p* < 0.001. KD: knockdown; CCK-8: Cell Counting Kit-8.

To elucidate the functional role of CCZ1 in CSCC cells, we used CCZ1-siRNA to decrease CCZ1 protein expression in ME180 and SiHa cells (Figure 4A). CCK-8 assays demonstrated that CCZ1 knockdown significantly suppressed the proliferation of ME180 and SiHa cells (Figure 4B). The downregulation of CCZ1 expression significantly attenuated the colony formation capacity of ME180 and SiHa cells (Figure 4C). Flow cytometry revealed that the downregulation of CCZ1 expression significantly increased the proportion of cells in the G2/M phase (Figure 4D). To elucidate the promoting effect of CCZ1 on CSCC cells in vivo, we utilized the sh-CCZ1 group (*n* = 4) and sh-scramble group (*n* = 4) of SiHa cells to subcutaneously inoculate nude mice. Tumor volumes and weights were notably lower in the sh-CCZ1 group compared to the sh-scramble group, and no significant difference in body weight was observed between the two groups (Figure 4E). Transwell assays revealed that ME180 and SiHa cells subjected to CCZ1 knockdown exhibited decreased migration and invasion abilities (Figure 4F). These findings indicated that CCZ1 promoted CSCC progression in vitro and in vivo.

### 3.5. Functional Enrichment Analysis of CCZ1 in CSCC

**Figure 5:**
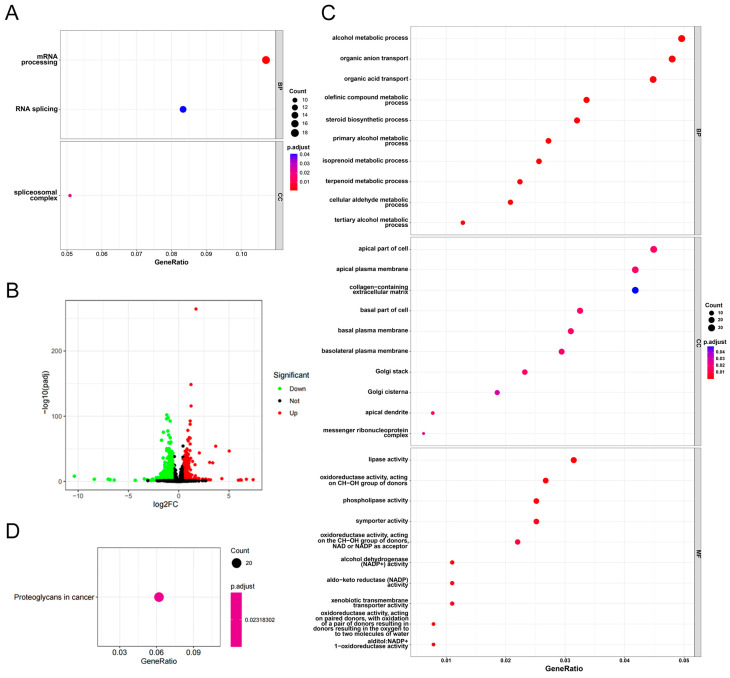
Functional enrichment analysis of CCZ1 in CSCC. (**A**) GO analysis of CCZ1 expression-related genes using CSCC expression data from TCGA database. (**B**) A volcano plot of differential gene expression between SiHa non-silencing and SiHa CCZ1-siRNA cells. (**C**) GO analysis of downregulated differentially expressed genes. (**D**) KEGG pathway analysis of the downregulated differentially expressed genes. CSCC: cervical squamous cell carcinoma; TCGA: The Cancer Genome Atlas; GO: Gene Ontology; KEGG: Kyoto Encyclopedia of Genes and Genomes.

To further elucidate the downstream pathways regulated by CCZ1 in CSCC cells, we performed GO analysis on CCZ1 expression-related genes using CSCC expression data from the TCGA database. The analysis revealed significant enrichment in pathways related to “mRNA processing,” “RNA splicing,” and the “spliceosomal complex” (Figure 5A). RNA-Seq analysis identified 267 differentially expressed genes (Log_2_|fold change| > 1, *p* < 0.05) (Figure 5B). Among these genes, 70.79% (189) were significantly downregulated in SiHa CCZ1-siRNA cells compared to SiHa non-silencing cells. GO analysis indicated that the downregulated genes were associated with several pathways, including “alcohol metabolic process”, “apical part of cell,” and “lipase activity” (Figure 5C). KEGG pathway analysis revealed the involvement of the downregulated genes in the “proteoglycans in cancer” pathway (Figure 5D). According to the GO analysis, the upregulated genes were linked to other pathways, including “organelle fission”, “spindle”, and “tubulin binding” (Figure S2A). KEGG pathway analysis revealed that the upregulated genes were involved in the “cell cycle” and “motor proteins” pathways (Figure S2B). These results indicate that CCZ1 in CSCC cells influences multiple signaling pathways.

### 3.6. CCZ1 KD Downregulated MMP2/MMP17 Expression

**Figure 6:**
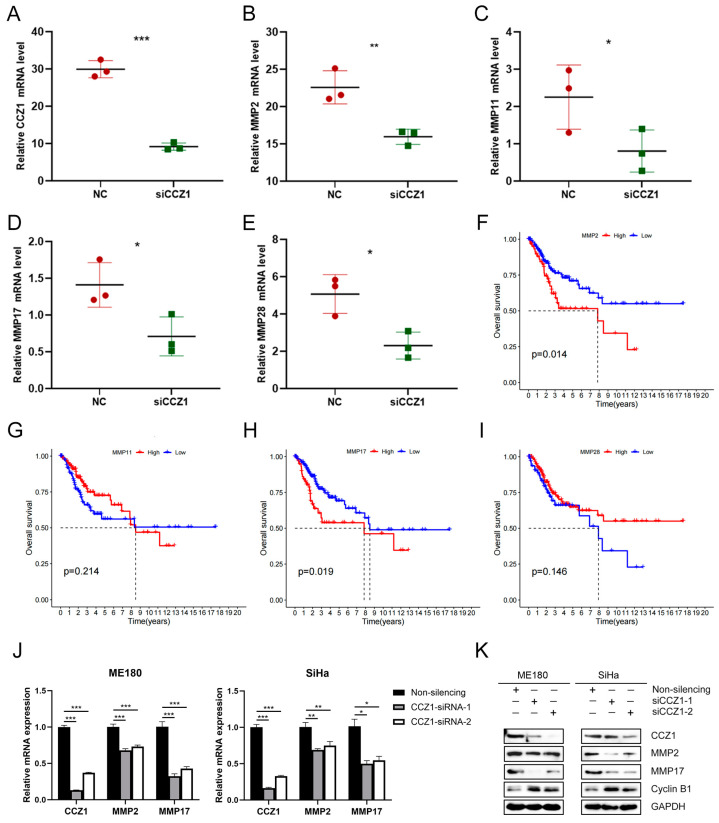
CCZ1 KD downregulated MMP2/MMP17 expression. (**A**–**E**) RNA-Seq analysis of the mRNA levels of CCZ1 (**A**), MMP2 (**B**), MMP11 (**C**), MMP17 (**D**), and MMP28 (**E**) in SiHa non-silencing and SiHa CCZ1-siRNA cells. (**F**–**I**) The Kaplan–Meier survival curves of the MMP2 (**F**), MMP11 (**G**), MMP 17 (**H**), and MMP28 (**I**) high groups and low groups. (**J**) qRT-PCR analysis of ME180 and SiHa cells transfected with CCZ1 siRNA or non-silencing siRNA. (**K**) Western blot analysis of ME180 and SiHa cells transfected with CCZ1 siRNA or non-silencing siRNA. * *p* < 0.05, ** *p* < 0.01, *** *p* < 0.001. CSCC: cervical squamous cell carcinoma; qRT-PCR: quantitative real-time PCR.

To examine the potential regulatory impact of CCZ1 on the expression of MMP protein family members, we used the aforementioned RNA sequencing data for analysis. Knockdown of CCZ1 significantly reduced the expression levels of CCZ1 (*p* < 0.001, Figure 6A), MMP2 (*p* < 0.01, Figure 6B), MMP11 (*p* < 0.05, Figure 6C), MMP17 (*p* < 0.05, Figure 6D), and MMP28 (*p* < 0.05, Figure 6E). To investigate the association between the mRNA levels of MMP family members and the prognosis of CSCC patients, we performed a Kaplan–Meier survival curve analysis. The results revealed that patients with high expression of MMP2 and MMP17 had a significantly poorer prognosis (*p* = 0.014 and *p* = 0.019, respectively; Figure 6F,H). However, the expression of MMP11 and MMP28 did not affect the OS of CSCC patients (*p* = 0.214 and *p* = 0.146, respectively; Figure 6G,I). Hence, we further investigated the relationship between CCZ1 and the expression of MMP2 and MMP17 in CSCC cell lines. CCZ1 knockdown in ME180 and SiHa cells decreased the mRNA and protein levels of MMP2 and MMP17 (Figure 6J,K). CCZ1 knockdown resulted in the upregulation of cyclin B1 protein expression (Figure 6K). Cyclin B1 is a well-established cell cycle checkpoint protein that regulates cell cycle checkpoints, facilitates G2/M phase transition, and accelerates cell cycle progression [37]. These results are consistent with previous experimental findings that CCZ1 knockdown promoted the transition of CSCC cells to the G2/M phase. These findings suggest that CCZ1 regulates the expression of MMP2 and MMP17 and is associated with a poor prognosis in patients with CSCC.

### 3.7. CCZ1 KD Inhibited CSCC Progression by Downregulating MMP2 and MMP17 Expression

**Figure 7:**
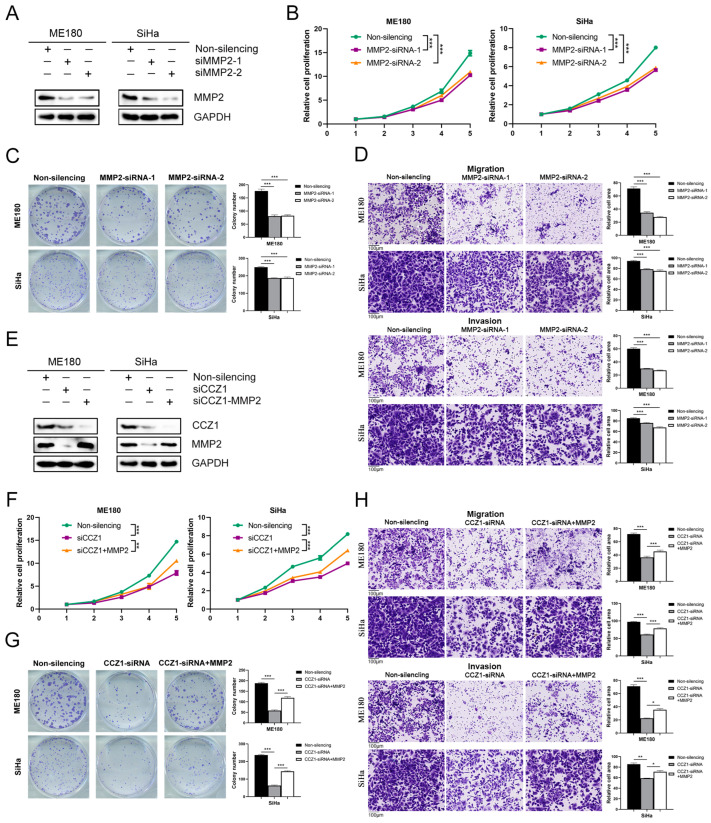
CCZ1 KD inhibited CSCC progression by downregulating MMP2 expression. (**A**) Western blot analysis of MMP2 expression in ME180 cells and SiHa cells transfected with MMP2 siRNA or non-silencing siRNA. (**B**) Cell proliferation was detected using the CCK-8 assay. (**C**) Cell colony formation assay. (**D**) Transwell assay for migration and invasion capability analysis. (**E**) Western blot analysis of MMP2 expression in ME180 cells and SiHa cells transfected as indicated. (**F**) Cell proliferation was detected using the CCK-8 assay. (**G**) Cell colony formation assay. (**H**) Transwell assay for migration and invasion capability analysis. * *p* < 0.05, ** *p* < 0.01, *** *p* < 0.001. KD: knockdown; CSCC: cervical squamous cell carcinoma; CCK-8: Cell Counting Kit-8.

**Figure 8:**
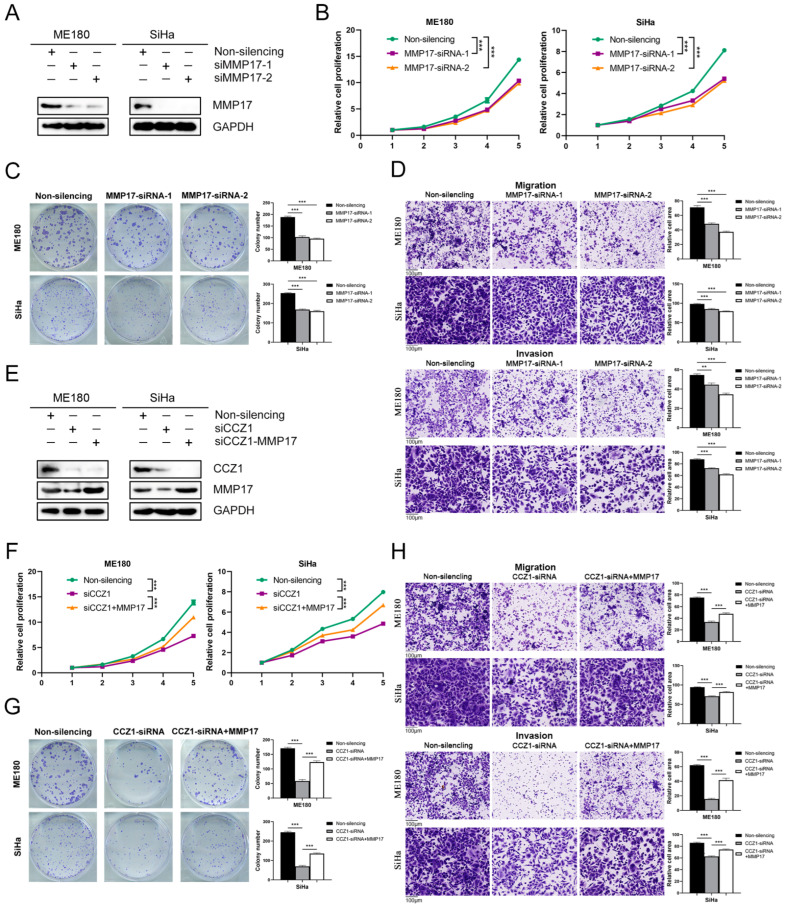
CCZ1 KD inhibited CSCC progression by downregulating MMP17 expression. (**A**) Western blot analysis of MMP17 expression in ME180 cells and SiHa cells transfected with MMP17 siRNA or non-silencing siRNA. (**B**) Cell proliferation was detected using the CCK-8 assay. (**C**) Cell colony formation assay. (**D**) Transwell assay for migration and invasion capability analysis. (**E**) Western blot analysis of MMP17 expression in ME180 cells and SiHa cells transfected as indicated. (**F**) Cell proliferation was detected using the CCK-8 assay. (**G**) Cell colony formation assay. (**H**) Transwell assay for migration and invasion capability analysis. ** *p* < 0.01, *** *p* < 0.001. KD: knockdown; CSCC: cervical squamous cell carcinoma; CCK-8: Cell Counting Kit-8.

To investigate the functional role of MMP2 and MMP17 in CSCC cells, we used MMP2-siRNA and MMP17-siRNA, respectively, to decrease protein expression levels in ME180 and SiHa cells (Figure 7A and Figure 8A). The downregulation of MMP2 and MMP17 expression significantly suppressed the proliferation (Figure 7B and Figure 8B), colony formation (Figure 7C and Figure 8C), and migration and invasion (Figure 7D and Figure 8D) of ME180 and SiHa cells. The effects of MMP2 and MMP17 knockdown in CSCC cells were consistent with CCZ1 knockdown. To investigate whether CCZ1 promoted the progression of CSCC by regulating the expression of MMP2 and MMP17, we restored MMP2 or MMP17 expression following CCZ1 knockdown in ME180 and SiHa cells (Figure 7E and Figure 8E). The restoration of MMP2 and MMP17 expression rescued the decreases in proliferation capacity (Figure 7F and Figure 8F), colony formation capacity (Figure 7G and Figure 8G), and migration and invasion (Figure 7H and Figure 8H) caused by CCZ1 knockdown in ME180 and SiHa cells. These data suggest that CCZ1 promotes CSCC cell proliferation, colony formation, migration, and invasion by upregulating the expression of MMP2 and MMP17.

## 4. Discussion

The present study investigated the expression pattern and prognostic significance of CCZ1 in CSCC. Our analysis of TCGA datasets revealed a significant upregulation of CCZ1 mRNA in CSCC samples compared to normal cervical tissues. Immunohistochemical analysis of clinical specimens revealed significantly greater CCZ1 protein expression in CSCC tumor tissues compared to normal cervical tissues. This observation supports the potential role of CCZ1 in CSCC pathogenesis. Notably, our findings indicate a robust association between elevated CCZ1 expression and poor prognosis in CSCC patients. Kaplan–Meier survival analysis demonstrated that patients with high CCZ1 expression had significantly shorter OS compared to patients with low CCZ1 expression. Subgroup analyses based on age and grade further supported the prognostic significance of CCZ1 and revealed poorer outcomes in patients with high CCZ1 expression across various clinical strata. Our study further elucidated the independent prognostic significance of CCZ1 in CSCC. Univariate and multivariate Cox regression analyses demonstrated that CCZ1 expression was an independent risk factor for CSCC prognosis. These findings highlight the potential utility of CCZ1 as a novel prognostic biomarker for CSCC and offer valuable prognostic information beyond traditional clinicopathological parameters. To enhance clinical applicability, we developed and validated a nomogram that integrated CCZ1 expression with age, stage, and tumor grade. The nomogram exhibited excellent predictive performance, which supports its potential as a reliable tool for individualized prognostication and treatment decision-making in CSCC patients. Although no studies have linked CCZ1 to tumors, existing research highlights its involvement in essential cellular processes, such as autophagy, intracellular material transport, and endosome maturation [10,12,13,14]. Our study highlights CCZ1 as a potential biomarker for the prognosis prediction of CSCC. However, our study did not address its influence on CSCC tumor cell processes, such as autophagy or material transport, which is a notable omission in our research.

Functional enrichment analysis provided insights into the biological processes associated with CCZ1 dysregulation in CSCC. Our results revealed significant enrichment of genes related to developmental processes and immune system-related pathways in the high-CCZ1-expression group, which supports potential roles for CCZ1 in CSCC progression via the modulation of these pathways. Correlation analysis revealed a positive association between CCZ1 expression and the expression of immunogenic cell death modulators, which indicates that CCZ1 is involved in the regulation of immune responses within the tumor microenvironment. Previous studies demonstrated the induction of immunogenic cell stress in HPV-associated malignancies following radiotherapy and cisplatin-based chemotherapy [38]. The construction of a gene set associated with immunogenic cell death predicted prognosis and the anti-tumor immune response in cervical cancer patients [39]. The present study examined the correlation between elevated CCZ1 expression in CSCC and the presence of immunogenic cell death modulators. These findings elucidate the multifaceted role of CCZ1 in CSCC pathobiology and support its potential as a therapeutic target.

Experimental validation using in vitro and in vivo models corroborated the functional significance of CCZ1 in CSCC progression. CCZ1 knockdown significantly attenuated the proliferation, colony formation, migration, and invasion of CSCC cells, which supports its role in the driving of tumorigenic phenotypes. CCZ1 knockdown also promoted cell cycle transition to the G2/M phase, which was consistent with the increased expression of cyclin B1. The dysregulation of cyclin B1 is linked to the progression of various cancers [40,41,42]. Our findings revealed a mechanistic link between CCZ1 and MMPs, particularly MMP2 and MMP17, which are known regulators of tumor invasion and metastasis. CCZ1 knockdown downregulated MMP2 and MMP17 expression, which provides mechanistic insights into the CCZ1-mediated promotion of CSCC progression. Investigations into the roles of MMPs in cancer have shown significant progress in recent years. MMPs have garnered increasing attention due to their established roles in tumor invasion, metastasis, and angiogenesis [17]. Studies of the expression and function of MMPs in cancer have provided valuable insights into their involvement in key processes driving tumor progression, including extracellular matrix remodeling and tumor microenvironment modulation [23,43]. Research on MMPs has provided insights into their potential as therapeutic targets for various cancers. Preclinical studies of MMP inhibitors and targeting strategies have shown promising results in suppressing cancer progression and metastasis [44]. However, challenges related to specificity, efficacy, and off-target effects have prompted ongoing efforts to refine MMP-targeted therapies for clinical translation. Investigations of the interplay between CCZ1 and MMPs have revealed intricate regulatory networks governing CSCC progression. CCZ1-mediated upregulation of specific MMPs, such as MMP2 and MMP17, has been implicated in the promotion of CSCC invasion and metastasis, which further supports the therapeutic potential of targeting these molecules in CSCC management.

Despite the significant insights gained from our investigation, several limitations of this study warrant acknowledgment. The significant discrepancy in the number of normal cervical tissue samples (*n* = 3) compared to CSCC samples (*n* = 239) is a limitation of our study. This imbalance may affect the robustness of our conclusions, as the small sample size of normal tissues might not fully capture the biological variability present in the general population. However, it is important to note that these cohort data come from the TCGA database, which inherently features this data distribution. To enhance the persuasiveness of our conclusions, we further verified the findings at the RNA and protein levels in an independent clinical cohort. Future studies should aim to include a larger number of normal tissue samples to validate our findings and ensure a more comprehensive comparison. Our study identified CCZ1 as a promising biomarker for CSCC prognosis, but its functional mechanisms in CSCC progression are not completely understood. Further mechanistic studies elucidating the molecular pathways of CCZ1 in CSCC pathogenesis are warranted. Although our experimental validation provided evidence of the functional significance of CCZ1 in vitro and in vivo, additional studies using larger sample sizes and more diverse experimental models are necessary to validate our findings. Our study focused on the role of CCZ1 in CSCC, but it is essential to recognize the complex interaction of multiple molecular players and pathways involved in CSCC progression. Future investigations examining the interactions of CCZ1, MMPs, and other key regulators of CSCC may provide a more comprehensive understanding of CSCC pathobiology.

## 5. Conclusions

CCZ1 is significantly overexpressed in CSCC, and higher CCZ1 expression is associated with poorer outcomes. CCZ1 promotes CSCC cell proliferation, colony formation, migration, and invasion by upregulating MMP2 and MMP17 expression, which supports its potential as a therapeutic target for CSCC treatment. These findings highlight the need for further research into CCZ1 inhibitors as a promising therapeutic approach. Researchers and clinicians should prioritize developing and testing CCZ1-targeted therapies to improve outcomes for patients with CSCC. 

## Data Availability

The original contributions presented in the study are included in the article/Supplementary Materials; further inquiries can be directed to the corresponding author.

## Supplementary Materials

The following supporting information can be downloaded at: https://www.mdpi.com/article/10.3390/biomedicines12071468/s1, Figure S1: CCZ1 mRNA was elevated in various tumor tissues; Figure S2: Functional enrichment analysis of CCZ1 in CSCC; Table S1: The information of the clinical cohort; Table S2: The sequences of siRNAs and shRNA.

## Abbreviations

CC: cervical cancer; CSCC: cervical squamous cell carcinoma; CAde: cervical adenocarcinoma; CIN: cervical intraepithelial neoplasia; HPV: human papillomavirus; GEF: guanine exchange factor; RAB7: member RAS oncogene family; GDP: guanosine diphosphate; GTP: guanosine triphosphate; MMP: matrix metalloproteinase; ECM: extracellular matrix; EGFR: epidermal growth factor receptor; VEGF: vascular endothelial growth factor; PD-1: programmed death-1; PD-L1: programmed death-ligand 1; TCGA: The Cancer Genome Atlas; UCSC: University of California, Santa Cruz; FPKM: fragments per kilobase of transcript per million mapped reads; FFPE: Formalin-fixed Paraffin-embedded; CHOL: cholangiocarcinoma; GBM: glioblastoma multiforme; PAAD: pancreatic adenocarcinoma; THYM: thymoma; KIRP: kidney renal papillary cell carcinoma; DLBC: lymphoid neoplasm diffuse large B-cell lymphoma; GSEA: Gene Set Enrichment Analysis; NES: normalized enrichment score; NOM: nominal; FDR: false discovery rate; GO: Gene Ontology; KEGG: Kyoto Encyclopedia of Genes and Genomes; PCRC: Cell Resource Center, Peking Union Medical College; siRNA: small interfering RNA; shRNA: short hairpin RNA; CCK-8: Cell Counting Kit-8; IHC: immunohistochemistry; OS: overall survival; PFS: progression -free survival; FIGO: The International Federation of Gynecology and Obstetrics; HR: hazard ratio; KD: knockdown.
