# Supplementary material for: CCZ1 Accelerates the Progression of Cervical Squamous Cell Carcinoma by Promoting MMP2/MMP17 Expression
Source: Biomedicines. 2024 Jul 3;12(7):1468. doi: 10.3390/biomedicines12071468 (PMC11274717; doi:10.3390/biomedicines12071468)
Supplement: Supplementary file 1 [file biomedicines-12-01468-s001.zip › Figure S1.pdf]

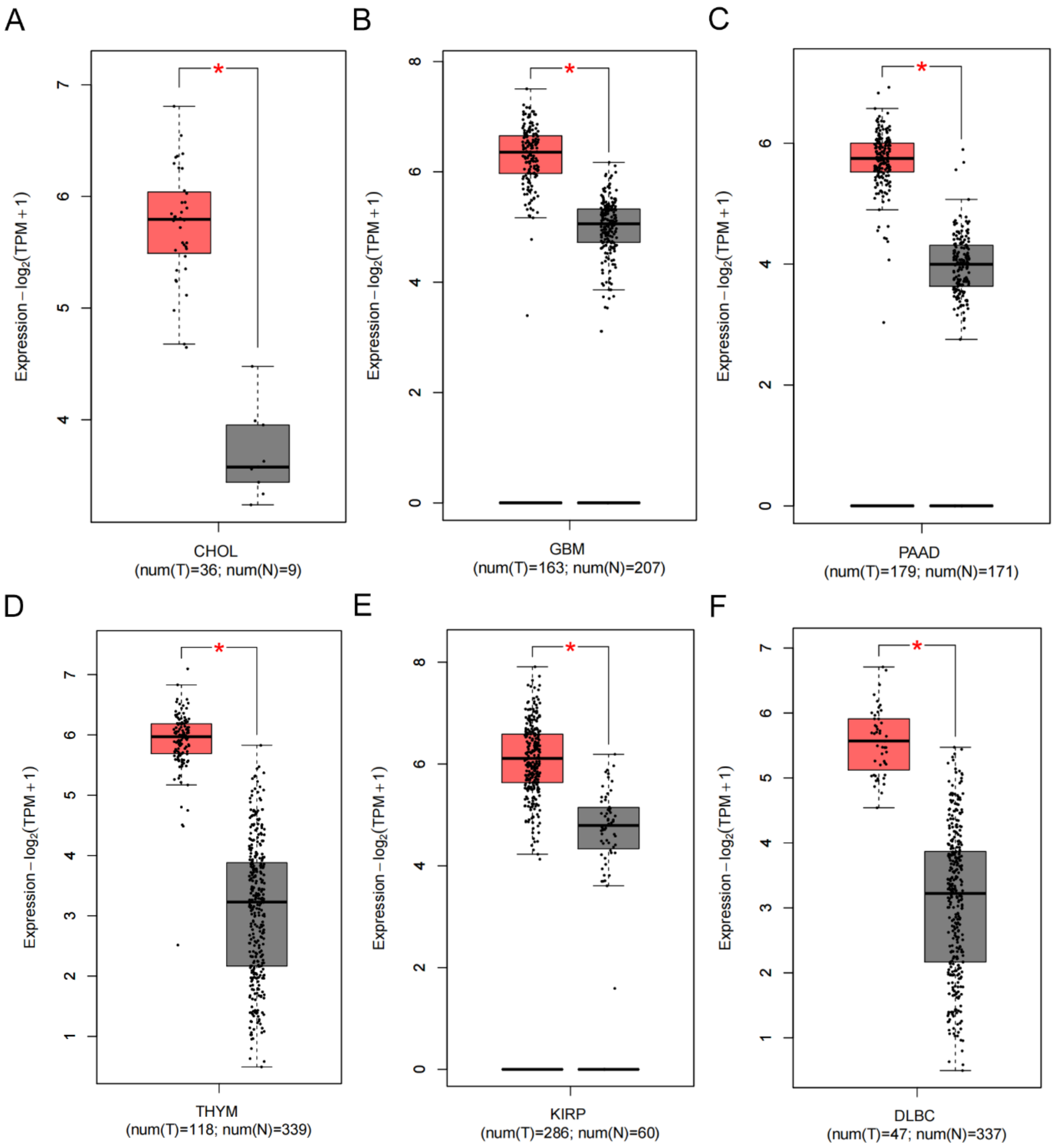

Figure S1: CCZ1 mRNA was elevated in various tumor tissues. (**A-F**) Abnormally high CCZ1 mRNA levels in CHOL (**A**), GBM (**B**), PAAD (**C**), THYM (**D**), KIRP (**E**), and DLBC (**F**) tissues. CHOL: cholangiocarcinoma; GBM: glioblastoma multiforme; PAAD: pancreatic adenocarcinoma; THYM: thymoma; KIRP: kidney renal papillary cell carcinoma; DLBC: lymphoid neoplasm diffuse large B-cell lymphoma. * $p < 0.05$. CHOL: cholangiocarcinoma; GBM: glioblastoma multiforme; PAAD: pancreatic adenocarcinoma; THYM: thymoma; KIRP: kidney renal papillary cell carcinoma; DLBC: lymphoid neoplasm diffuse large B-cell lymphoma.
