# Supplementary material for: CCZ1 Accelerates the Progression of Cervical Squamous Cell Carcinoma by Promoting MMP2/MMP17 Expression
Source: Biomedicines. 2024 Jul 3;12(7):1468. doi: 10.3390/biomedicines12071468 (PMC11274717; doi:10.3390/biomedicines12071468)
Supplement: Supplementary file 1 [file biomedicines-12-01468-s001.zip › Figure S2.pdf]

A

B

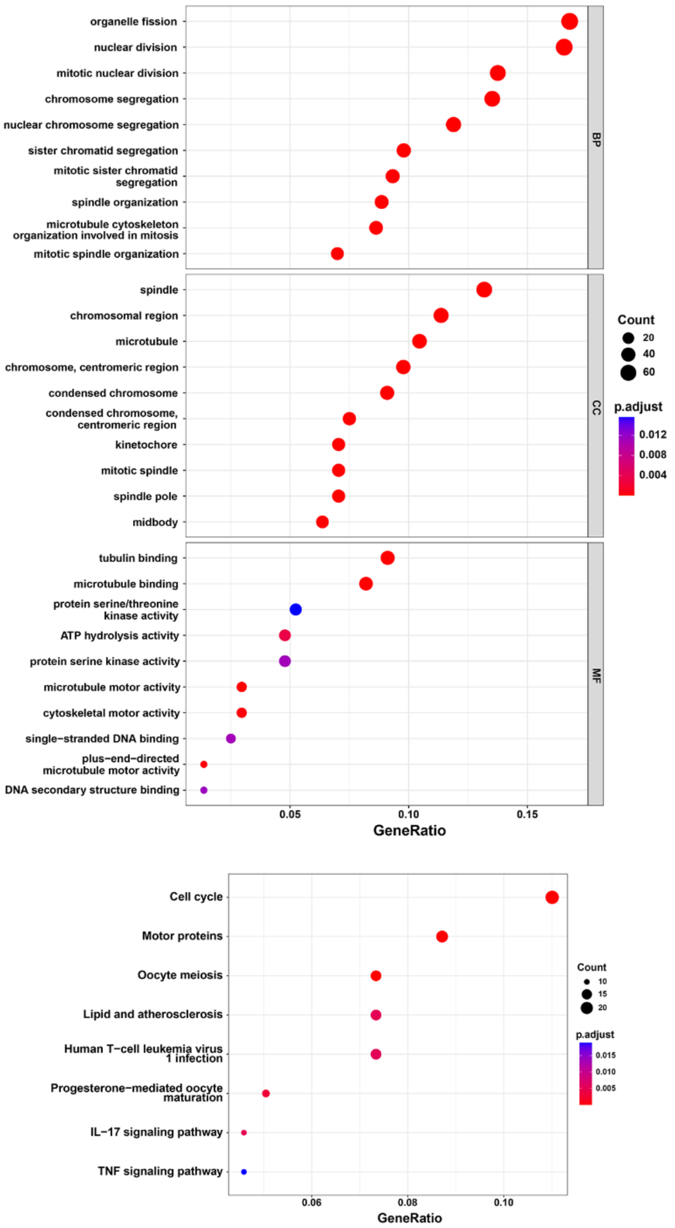

Figure S2: Functional enrichment analysis of CCZ1 in CSCC. (A) GO analysis of upregulated differentially expressed genes. (B) KEGG pathway analysis of the upregulated differentially expressed genes. CSCC: cervical squamous cell carcinoma; GO: Gene Ontology; KEGG: Kyoto Encyclopedia of Genes and Genomes.
