# Supplementary material for: CCZ1 Accelerates the Progression of Cervical Squamous Cell Carcinoma by Promoting MMP2/MMP17 Expression
Source: Biomedicines. 2024 Jul 3;12(7):1468. doi: 10.3390/biomedicines12071468 (PMC11274717; doi:10.3390/biomedicines12071468)
Supplement: Supplementary file 1 [file biomedicines-12-01468-s001.zip › Table S2.pdf]

Table S2: The sequences of siRNAs and shRNA.

**siRNA or shRNA**	**Sequence (5′-3′)**
Non-Silencing	UUCUCCGAACGUGUCACGUTT
CCZ1-siRNA-1	UGAAGAUGAGGAGAUCAUUTT
CCZ1-siRNA-2	AGGACAUUUAGCCCAUCAATT
MMP2-siRNA-1	UGUGUUCUUUGCAGGGAAUTT
MMP2-siRNA-2	UGAAGGACACACUAAAGAATT
MMP17-siRNA-1	CCACAAUGACAGGACUUAUTT
MMP17-siRNA-2	UCAUGUACUACGCCCUCAATT
CCZ1-shRNA	ATGAAGATGAGGAGATCATTG
