# Supplementary figures and images for: CCZ1 Accelerates the Progression of Cervical Squamous Cell Carcinoma by Promoting MMP2/MMP17 Expression
Source: Biomedicines. 2024 Jul 3;12(7):1468. doi: 10.3390/biomedicines12071468 (PMC11274717; doi:10.3390/biomedicines12071468)

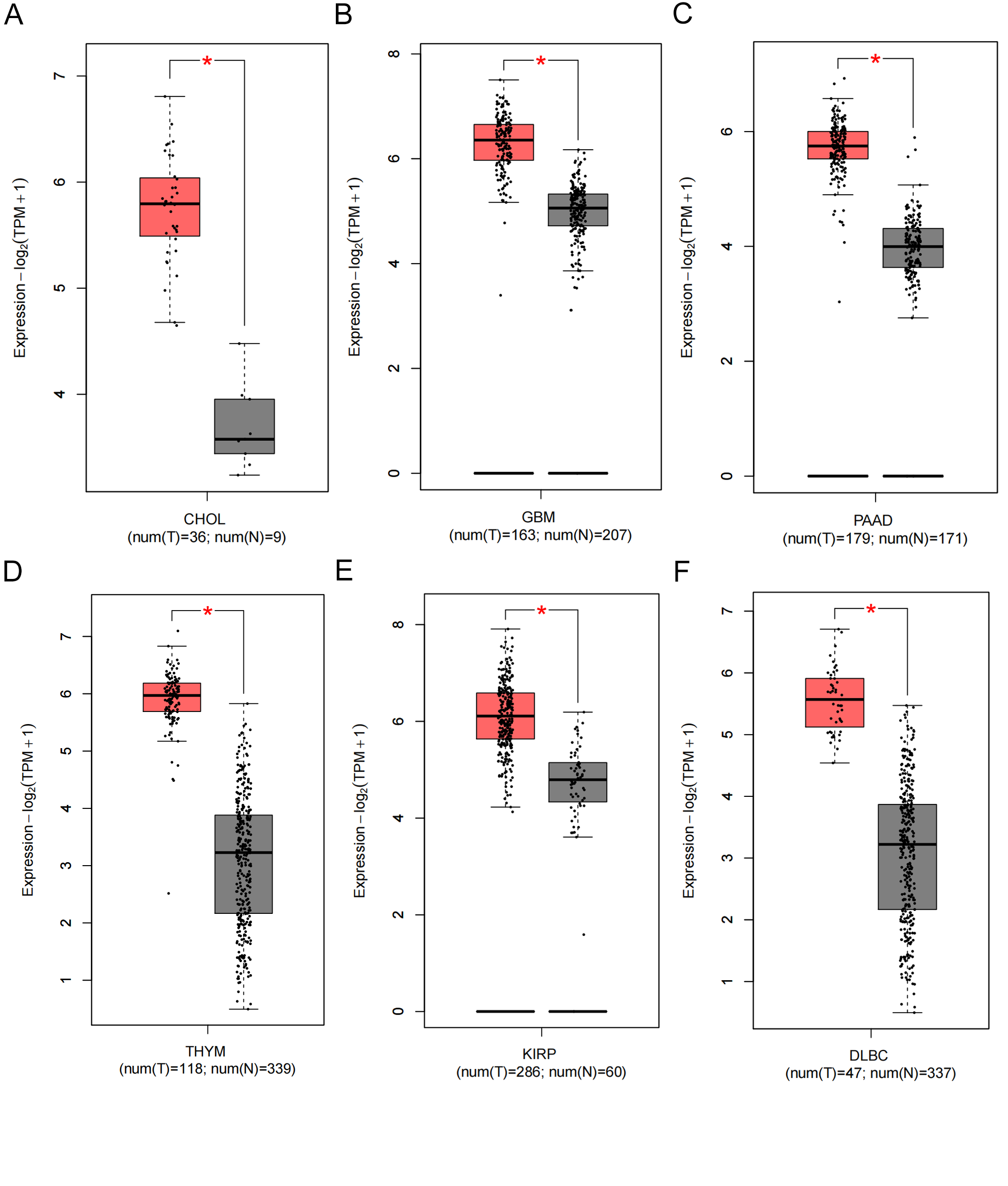

Supplement: Supplementary file 1 [file biomedicines-12-01468-s001.zip › Figure S1.tif]
